# The Video Head Impulse Test and the Influence of Daily Use of Spectacles to Correct a Refractive Error

**DOI:** 10.3389/fneur.2018.00125

**Published:** 2018-03-07

**Authors:** T.S. van Dooren, F.M.P. Lucieer, A.M.L. Janssen, H. Kingma, R. van de Berg

**Affiliations:** ^1^Division of Balance Disorders, Department of Otorhinolaryngology and Head and Neck Surgery, Faculty of Health Medicine and Life Sciences, School for Mental Health and Neuroscience, Maastricht University Medical Centre, Maastricht, Netherlands; ^2^Department of ENT/Audiology, School for Mental Health and Neuroscience (MHENS), Maastricht University Medical Centre, Maastricht, Netherlands; ^3^Department of Methodology and Statistics, Care and Public Health Research Institute (CAPHRI), Maastricht University, Maastricht, Netherlands; ^4^Faculty of Physics, Tomsk State University, Tomsk, Russia

**Keywords:** Video Head Impulse Test, Head Impulse Test, vestibulo-ocular reflex, VOR, refractive error, Diopter, spectacles

## Abstract

**Objective:**

To determine the influence of daily use of spectacles to correct a refractive error, on the vestibulo-ocular reflex (VOR) gain measured with the video head impulse test (vHIT).

**Study design:**

This prospective study enrolled subjects between 18 and 80 years old with and without a refractive error. Subjects were classified into three groups: (1) contact lenses, (2) spectacles, and (3) control group without visual impairment. Exclusion criteria comprised ophthalmic pathology, history of vestibular disorders, and alternated use of spectacles and contact lenses in daily life. Corrective spectacles were removed seconds before testing. One examiner performed all vHIT’s under standardized circumstances using the EyeSeeCam system. This system calculated the horizontal VOR gain for rightward and leftward head rotations separately.

**Results:**

No statistically significant difference was found in VOR gain between the control group (*n* = 16), spectacles group (*n* = 48), and contact lenses group (*n* = 15) (*p* = 0.111). Both the spectacles group and contact lenses group showed no statistically significant correlation between VOR gain and amount of refractive error, for rightwards (*p* = 0.071) and leftwards (*p* = 0.716) head rotations. There was no statistical significant difference in VOR gain between testing monocularly or binocularly (*p* = 0.132) and between testing with or without wearing contact lenses (*p* = 0.800).

**Conclusion:**

In this study, VOR gain was not influenced by wearing corrective spectacles or contact lenses on a daily basis. Based on this study, no corrective measures are necessary when performing the vHIT on subjects with a refractive error, regardless of the way of correction.

## Introduction

The vestibulo-ocular reflex (VOR) enables gaze stabilization during head movements with an instant compensatory eye movement in the direction opposite to the head movement (1). The VOR can be used to assess vestibular function (2). A test to examine the VOR in the high frequency domain is the Head Impulse Test (HIT). The HIT comprises a passive, unpredictable, low-amplitude, rapid head rotation, performed by an examiner, while the patient maintains gaze on a target (2–4). In case of a peripheral vestibular loss, the eyes will not be able to maintain gaze on the target and are forced to make a compensatory catch-up saccade. Saccades can occur after the head movement (“overt” saccades), or during the head movement (“covert” saccades). Even for experts, covert saccades are often undetectable by the naked eye during the manual HIT (5–7).

Covert saccades can be detected by the Video Head Impulse Test (vHIT). This test uses a lightweight video-oculography device with a high-speed infrared video camera while performing the HIT. The camera tracks head and pupil movement during the head impulse and, therefore, detects both overt and covert saccades (8). The vHIT is a validated method to assess peripheral vestibular dysfunction in the high frequency domain (9, 10). Unlike the scleral coil method, it is noninvasive and easy for clinical use (7, 11). At this moment, the VOR gain is considered to be the main outcome parameter to measure performance. It represents the correlation between eye velocity and head velocity, and can be calculated in various ways (4, 8, 12–14).

Regardless of the way of calculation, the VOR gain is influenced by eye movements. When wearing optical devices, the eye movement will change in order to assure gaze stabilization. This mechanism can be so extreme that total reversal of the direction of the VOR was observed during a study with dove prisms (15). A more modest change takes place when wearing spectacles to correct a refractive error. Due to the prism effect of the glass, objects are viewed in a different line of sight than the principal axis of the lens of the eye. This means, in comparison to “normal” vision, a bigger or smaller eye movement is needed to maintain gaze stabilization while wearing corrective spectacles. The difference in eye movement depends on the diopter of the glass (1). A bigger or smaller eye movement during the same head movement means a smaller of bigger VOR gain value.

In this study, it was hypothesized that the VOR gain, as measured with the vHIT, could be influenced by wearing spectacles on a daily basis to correct a refractive error. Wearing contact lenses would not influence VOR gain at any degree because the contact lenses rotate along with the eyes and, therefore, have no prism effect (1, 16). This hypothesis might imply, when testing VOR gain with vHIT in subjects with a refractive error, corrective measures should be made to prevent false diagnosis of vestibular dysfunction, depending on the way of correction.

## Materials and Methods

This study determined the influence of daily use of spectacles to correct a refractive error, on the VOR gain measured with the vHIT. Subjects wearing spectacles were compared to subjects without visual impairment and subjects wearing contact lenses.

### Study Population

A prospective study was performed on volunteers in optician stores in Maastricht. These settings were chosen in order to precisely determine the diopter in the worn spectacles and contact lenses for each subject. Subjects met the following inclusion criteria: (1) age between 18 and 80 years old, (2) presence of refractive error, and (3) wearing corrective spectacles or contact lenses on a daily basis. Subjects were excluded when they (1) alternated between glasses and contact lenses during daily life or when they (2) were unable to see the point of fixation for the vHIT. Further exclusion criteria comprised (3) ophthalmic pathology or surgery, (4) neck pathology, (5) history of vestibular disorders, and (6) a difference in refractive error between both eyes of more than 4 diopter.

The same exclusion criteria were applied to the control group, which comprised healthy volunteers between 18 and 80 years old with no visual impairment. Informed consent was obtained before testing.

### Protocol

The VOR gain can be influenced by artifacts resulting from goggle slippage, incorrect calibration, imperfect pupil tracking, blinking, head overshoot, touching goggles, patient inattention, and target distance (8, 14, 17–19). To reduce these artifacts to a minimum, a strict testing protocol was designed by all authors and used by the examiner, as described below.

#### Experimental Setup

The examiner (TD) ensured a constant distance of 2 m from the back of the chair to the point of fixation. A static chair was used to prevent body movement during testing. The point of fixation consisted of a laser on a tripod, pointing a green dot on a white wall to create maximum contrast. The examiner adjusted the fixating point to the eye level of the subject. The light intensity was measured with an illuminance meter, and the light intensity at the focusing point in the room was kept between 80 and 320 lux. This ensured a small pupil in every subject and, therefore, facilitated a wider range for measuring the eye movements. At the same time, it minimized the change of artifacts due to light reflection onto the pupil.

#### vHIT Preparations

Since the amount of refractive error could differ between the eyes, the subjects left eye was covered with a sticker before the goggles were applied. By this, only the right eye was measured. As the Diopter of the correction (spectacles/contact lenses) influences the VOR, this value was used for inclusion, rather than the refractive error itself. Goggle movement was minimized by adjusting the strap of the goggles to every subject. The camera was focused on the pupil while the subject looked at the point of fixation with eyes wide open. In case the eyelids were in front of the pupil, the examiner adjusted the rim of the goggles so they would hold the eyelids back. A five-point laser grid, mounted on the goggles, was used to calibrate the EyeSeeCam system (EyeSeeCam VOG; Munich, Germany). It projected a red luminous dot pattern on the wall. The examiner instructed all subjects to look at five dots in the same order without moving their head. When vision was too impaired to see the red dots, the subjects were instructed to follow the examiners finger while the examiner pointed out these dots. The examiner assessed the quality of the calibration and determined whether the process needed to be repeated. After calibration, the subject was instructed to not touch (the strap of) the goggles, their face and/or their hair.

#### Video Head Impulse Test

One trained examiner (TD) performed the horizontal vHIT on every subject. The examiner stood behind the subject with both hands on top of the head, holding it firmly without touching the strap or goggles. Before the start of official testing, slow horizontal sinusoidal head movements were given in order to assess neck stiffness and to give final instructions. Subjects were instructed to relax their neck, keep their eyes wide open and fixate on the target in front of them. The examiner continuously repeated these instructions to facilitate optimal awareness of the subject. The head impulses comprised fast (peak velocity >150°/s) horizontal rotational head movements with a low amplitude (±20°), unpredictable in timing and direction (14). Only outward impulses were used (20).

#### Testing Paradigm

One recording session consisted of two trials with at least 10 impulses to each side in total. Every trial resulted in two VOR gain values as outcome, one for the head movement to the right and one for the head movement to the left. In total, every recording session consisted of four VOR-gain values. A stopwatch was used to time every recording session.

Subjects in the spectacles group underwent one recording session: without wearing their spectacles. Subjects in the contact lenses group underwent two recording sessions: with and without wearing contact lenses. This way, it was possible to evaluate the influence of wearing contact lenses during the vHIT. Subjects in the control group underwent three recording sessions: the first and third recording sessions were performed binocularly, the second session was performed monocularly with the left eye covered. This setup was used to determine the reproducibility of the trials and to determine the difference in outcomes between monocular (left eye covered) and binocular (no eye coverage) testing. All recording sessions were sequentially performed. Table 1 shows an overview of the testing paradigm as described above.

**Table 1:** Overview of the testing paradigm.

**Group 1: spectacles**				
*Trial 1*	VOR gain R	*Trial 2*	VOR gain R	= *Recording session 1*: testing without wearing spectacles
	VOR gain L		VOR gain L	
**Group 2: contact lenses**				
*Trial 1*	VOR gain R	*Trial 2*	VOR gain R	= *Recording session 1*: testing while wearing contact lenses
	VOR gain L		VOR gain L	
*Trial 3*	VOR gain R	*Trial 4*	VOR gain R	= *Recording session 2*: testing without wearing contact lenses
	VOR gain L		VOR gain L	
**Group 3: control**				
*Trial 1*	VOR gain R	*Trial 2*	VOR gain R	= *Recording session 1*: binocular testing
	VOR gain L		VOR gain L	
*Trial 3*	VOR gain R	*Trial 4*	VOR gain R	= *Recording session 2*: monocular testing
	VOR gain L		VOR gain L	
*Trial 5*	VOR gain R	*Trial 6*	VOR gain R	= *Recording session 3*: binocular testing
	VOR gain L		VOR gain L	

*Group 1 (spectacles) underwent one recording session: without wearing their spectacles. Group 2 (contact lenses) underwent two recording sessions: with and without wearing their contact lenses. All recording sessions in Group 1 and 2 were performed monocularly (with the left eye covered). Group 3 (control group) underwent three recording sessions: the first and third recording sessions were performed binocularly (without eye coverage) and the second session was performed monocularly (left eye covered). One recording session consisted of two trials with at least 10 impulses to each side in total, tested under the same conditions*.

*VOR = vestibulo-ocular reflex, R = rightwards head impulse, L = leftwards head impulse*.

### Data Analysis

The shapes of all traces were assessed in consensus by three of the authors (TD, FL, and RB). In order to detect artifacts and look for a possible correlation between artifacts and refractive error, data was blinded. During analysis, two trials of the same recording session were kept together as a pair. Every pair of trials was placed in one of the following subgroups: (1) phase lead (eyes peak velocities appeared >20 ms earlier than head peak velocity), (2) phase lead with overt saccades (≥50% of all the impulses on one side showed overt saccades), (3) small overt saccades (>50% of the overt saccades were slower than 100°/s), (4) large overt saccades (>50% of the overt saccades were faster than 100°/s), (5) backward overt saccades (overt saccades went into opposite direction), (6) noise (traces were not completely smooth but did not have saccades), and (7) normal. Only phase lead was considered to be an artifact that could influence VOR gain and for this reason subjects with a phase lead were excluded.

The EyeSeeCam software *(revision r3448M, April 2016)* backed by Matlab scripts was used for data analysis.

### Statistical Analysis

All statistical analysis were performed using IBM SPSS Statistics version 23. Normality was checked by the Shapiro–Wilk test and visual inspection of the outcome distribution. Where multiple comparisons were made, the Bonferroni adjusted *p*-values are given and compared to a standard *p*-value of 0.05. The VOR gain of every trial was calculated by the EyeSeeCam software for rightward and leftward head impulses separately. The Chi square test and one-way analysis of variance (ANOVA) were used to evaluate the homogenous nature of the groups (gender and age).

To evaluate the test–retest reliability as the consistency between the repeated measures of the same outcome condition, two-way random intraclass correlation coefficients were calculated for the repeated trials within one recording session (21). A two-way repeated measures ANOVA with 2 within-subject factors [side (left/right) and monocular testing (yes/no) or testing with contact lenses (yes/no)] was used to detect a statistically significant difference in testing monocularly (left eye covered) or binocularly (no eye coverage) and testing with or without wearing contact lenses.

The difference in VOR gain between the groups was analyzed with an ANOVA repeated measures with 1 within-subject factor (side) and 1 between-subject factor (group).

A regression analysis was used to determine the effect of Diopter and group (spectacles/contact lenses) on the VOR gain.

### Ethical Considerations

This study was performed in accordance with the guidelines outlined by Dutch legislation. According to the Medical Research Involving Human Subjects Act (WMO) ethical approval was not required, since the purpose of this study was to validate our own system and to obtain the normative values.

## Results

In total, 79 subjects were included. No significant difference was found in gender. A statistically significant difference in age was found between the spectacles group and the control group (*p* = 0.005) (Table 2). Subjects were wearing corrective spectacles for at least 4 months, up to 60 years.

**Table 2:** Baseline characteristics of the study population.

Characteristics	Spectacles	Contact lenses	Control	All
N	48	15	16	79
Male	26	5	7	38
Female	22	10	9	41
Mean age in years (SD)	54 (17)^a^	43 (10)	39 (14)^a^	48 (16.5)

*^a^A statistical significant difference in age between spectacles group and the control group (*p* = 0.005)*.

The first trial of the vHIT started within 90–300 s after removal of the correction. One recording session (Table 1) did not take longer than 480 s.

During visual assessment of the vHIT graphs no covert saccades were observed and no causality was seen between refractive errors and shape of the traces (as classified into the subgroups).

The trials within one recording session (Table 1) showed a good test–retest reliability, the intraclass correlation coefficient varied between 0.707 (*p* = 0.012) and 0.959 (*p* = 0.000).

There was no statistical significant difference in VOR gain between testing monocularly (left eye covered) or binocularly (no eye coverage) [*F*(1,15) = 2.538, *p* = 0.132], between testing with or without wearing contact lenses [*F*(1,14) = 0.067, *p* = 0.800] and between rightwards and leftwards head rotations [*F*(1,76) = 2.370, *p* = 0.128].

The VOR gain was compared between the spectacles group, contact lenses group, and control group. No statistically significant difference was found in VOR gain between these groups [*F*(2,76) = 2.265, *p* = 0.111]. Regarding the VOR gain for different Diopter, no significant interaction was found between group (spectacles/contact lenses) and Diopter for rightwards (*p* = 0.376) and leftwards (*p* = 0.189) head rotations. The spectacles group tended to show a positive relation between refractive error and VOR gain, but both in the spectacles group and contact lenses group no statistically significant correlation was found between VOR gain and different Diopter, for rightwards (*p* = 0.071) and leftwards (*p* = 0.716) head rotations.

**Figure 1:**
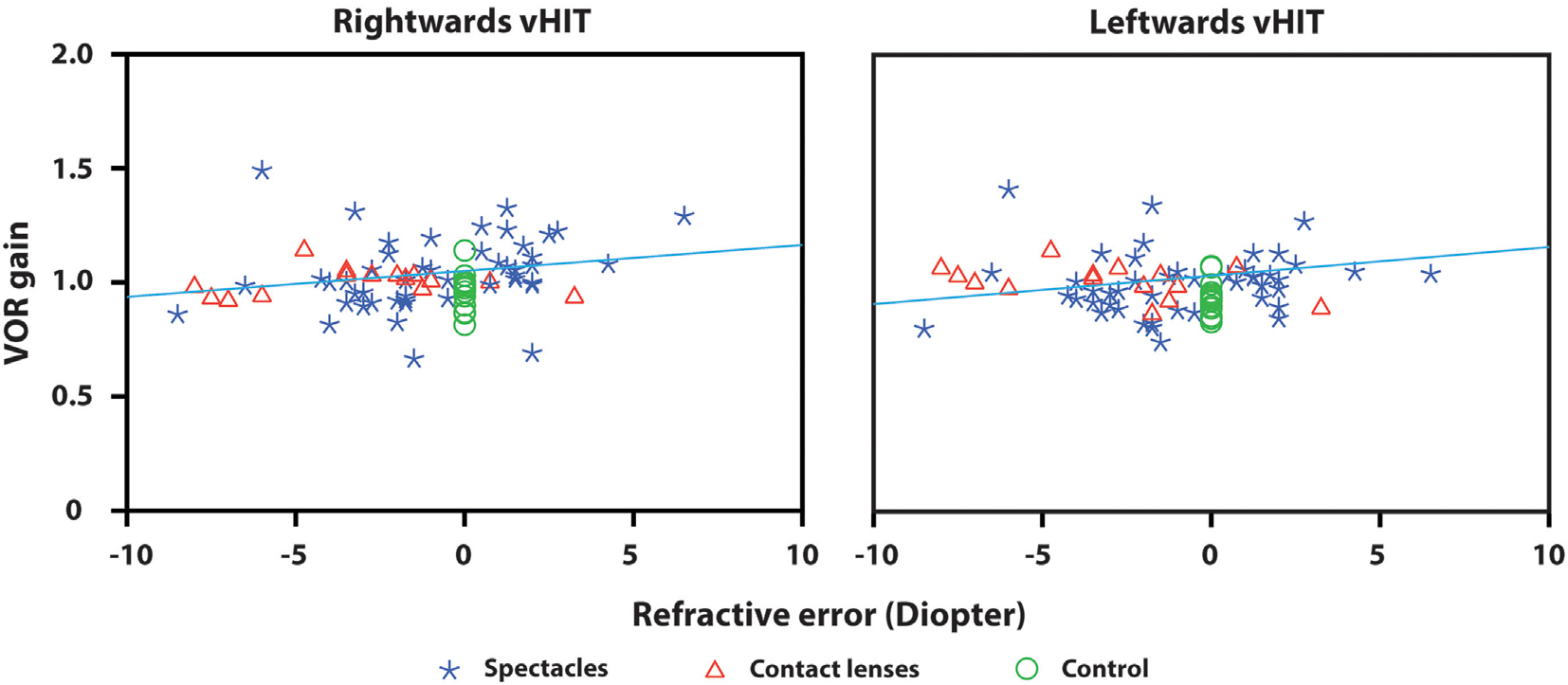
Vestibulo-ocular reflex gain plotted against refractive error for right- and leftwards head impulses. Every symbol represents the VOR gain of one subject calculated by the EyeSeeCam system. VOR gain did not differ significantly between the groups (spectacles, contact lenses, control group). No statistically significant correlation was found between VOR gain and different Diopter in both the spectacles group and contact lenses group for rightwards and leftwards head rotations. The regression line shows a tendency of positive relation between refractive error and VOR gain, but the difference is negligible and has no clinical significance.

Compared to the control group, VOR gain measured by the vHIT was not influenced by refractive error and daily use of spectacles or contact lenses (Figure 1).

## Discussion

To our knowledge, this is the first study to determine the influence of daily use of spectacles to correct a refractive error on the VOR gain measured by the vHIT, on a study population of this size. This study showed no statistically significant correlation between VOR gain and amount of refractive error and no significant difference was found in VOR gain between the groups (corrective spectacles, contact lenses, and control group without visual impairment). The spectacles group tended to show a positive relation between refractive error and VOR gain, but the effect size was negligible. This means vHIT is not influenced by Diopter or the way it is being corrected in daily life. Therefore, based on this study, no special measures are necessary when performing the vHIT on subjects with a refractive error.

Although this study illustrates that VOR gain is not influenced by a refractive error, regardless of the Diopter and way of correction, other studies did show VOR gain changes when exposed to sensory rearrangement such as magnifying spectacles or prisms (15–17, 22–24). The differences could be partly explained by the methods. First, none of the studies tested the VOR gain by using the vHIT. This implies that other methods were used that investigated different frequencies. Second, in some studies, subjects were tested in the dark or whilst wearing the temporary sensory rearrangements, in contrary to our subjects.

However, the discrepancy between this study and other studies could mainly be explained by two theories about centrally regulated mechanisms, as will now be described. The first mechanism is dual state adaptation. Dual state adaptation means the ability to switch between different states, for instance, between vision with corrective spectacles and normal vision (without sensory rearrangements). This adaptive process is enhanced by repeated exposure and results in adapting and readapting within seconds. In previous studies, subjects were exposed to the sensory rearrangement for only one or two periods of 40 min to a maximum of 4 weeks and tested immediately after. Our study population had been wearing corrective spectacles for at least 4 months, up to 60 years. It might be possible that these well exposed subjects benefited from an enhanced dual state adaptation and already readapted to vision without their spectacles within the 90–300 s between removal of the correction and start of the vHIT. As a result, no VOR gain change could be measured by the vHIT (16, 22, 24–27). The second theory could be central compensation by the brain. For example: in case of aniseikonia (a large difference in refractive error between both eyes), the brain is able to compensate for the distorted images that projected on the retina. The same compensation could be happening while wearing corrective spectacles. This would imply that the VOR would not change while wearing corrective spectacles and therefore will not influence the VOR gain as measured by the vHIT (28).

Regarding testing methods, no difference was found in VOR gain between monocularly and binocularly testing, and between testing with and without wearing contact lenses. Furthermore, it showed good reproducibility of the vHIT. This implies that VOR gain is not influenced by monocular testing, wearing contact lenses, and repeatedly testing the vHIT.

This study showed a non significant difference in VOR gain between rightwards en leftwards head rotations. At high head impulse accelerations, it was shown that the latency of the adducting eye is longer than the latency of the abducting eye accompanied with on average 15.3% higher gains of the adducting eye than gains of the abducting eye (29). As described in the methods, for practical reasons, we choose to detect and compare only eye movements of the right eye and only varied the visual fixation conditions during the head impulses (monocular or binocular fixation, with or without contact lenses). This implies that we anticipated on a maximum 15.3% higher gain of the VOR of the fastest impulses to the right.

One limitation of this study is the fact that the subjects in the control group were younger than the subjects of the spectacle group. Articles showed no VOR gain change until the age of 80 years, which means that it should not influence the outcome of this study, since subjects older than 80 years were excluded (3, 20, 30).

## Conclusion

Based on this study, corrective measures are not necessary when performing the vHIT on subjects with a refractive error, regardless of the way of correction.

## Ethics Statement

This study was performed in accordance with the guidelines outlined by Dutch legislation. According to the Medical Research Involving Human Subjects Act (WMO) ethical approval was not required, since the purpose of this study was to validate our own system and to obtain the normative values.

## Author Contributions

Design of the work, interpretation, revising the work, final approval of the version to be published, and agreement to be accountable for all aspects of the work in ensuring that questions related to the accuracy or integrity of any part of the work are appropriately investigated and resolved: HK, RB, TD, FL, and AJ. Acquisition: TD. Analysis: AJ and TD.

## Conflict of Interest Statement

The authors declare that the research was conducted in the absence of any commercial or financial relationships that could be construed as a potential conflict of interest. The reviewer JO and handling editor declared their shared affiliation.
